# Supplementary material for: Membrane fission via transmembrane contact
Source: Nat Commun. 2024 Mar 30;15:2793. doi: 10.1038/s41467-024-47122-w (PMC10981662; doi:10.1038/s41467-024-47122-w)
Supplement: Supplementary file 1 — Supplementary Information [file 41467_2024_47122_MOESM1_ESM.pdf]

Membrane fission via transmembrane contact

Spencer et al.

<u>Supplementary Information</u>

Supplementary Figures

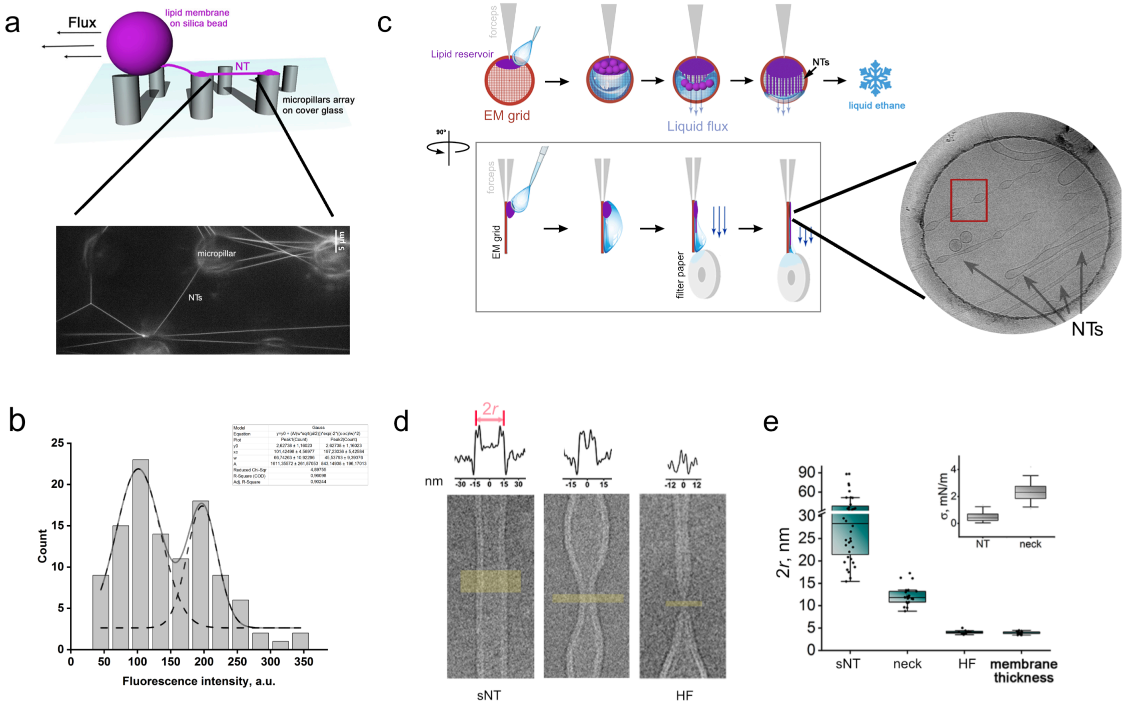

Supplementary Figure 1. Experimental methodologies for the formation and fission detection of membrane tubes. a. Fluorescence setup. 40 µm silica beads covered with multilamellae of the desired membrane composition are introduced into a microfluidic chamber containing a SU8 polymer micropillar array on a cover glass. The beads are "rolled" on top of the pillars by applying a microfluidic flow or carefully tilting the chamber (upper scheme). The resulting nanotubes (NTs) are freely suspended between the pillars (the lower image shows RhPE fluorescence). **b**. Distribution of integral fluorescence per unit length for tubes with the same membrane composition. The first peak corresponds to the population of SM tubes, while the second, with doubled intensity, corresponds to DM tubes[1]. **c.** CryoEM setup. Silica beads with membrane lamellae, as in (a), are deposited on a glow-discharged cryoEM grid. The beads can then be rolled on the grid by manual tilting. In that case, the freezing of the sample is delayed, and only tubes in a steady state can be observed. Alternatively, the beads or a lipid multilayer can be deposited on one side of the grid while tube extension is promoted by lipid flow induced by blotting of the grid on the opposite side (upper scheme). Freezing of the sample follows immediately upon blotting, allowing for the detection of remodelling intermediates. The lower micrograph illustrates the result of the later quasi-dynamical method with many nanotubes (NTs) undergoing constriction. Retraction or fission is detected in a 2 µm hole of the holey grid. **d**. Representative images of the three main membrane geometries as detected by cryoEM upon SM tube formation by the method depicted in c. The plot profiles correspond to the area marked in yellow for each structure. $2r$ corresponds to the distance between the external lipid heads of the NT (outer peaks of the corresponding plot profile), except in the case of HF, where it corresponds to the head-to-head distance of the transversal section of the intermediate. **e**. Quantification of the radii of the tubes and structures as in d. Each point corresponds to a separate structure or NT. Inset shows the values σ corresponding to the radii measured for the NT and the constricted neck and κ = 1.3 mN/m for a POPC membrane. Source data are provided as a Source Data file.

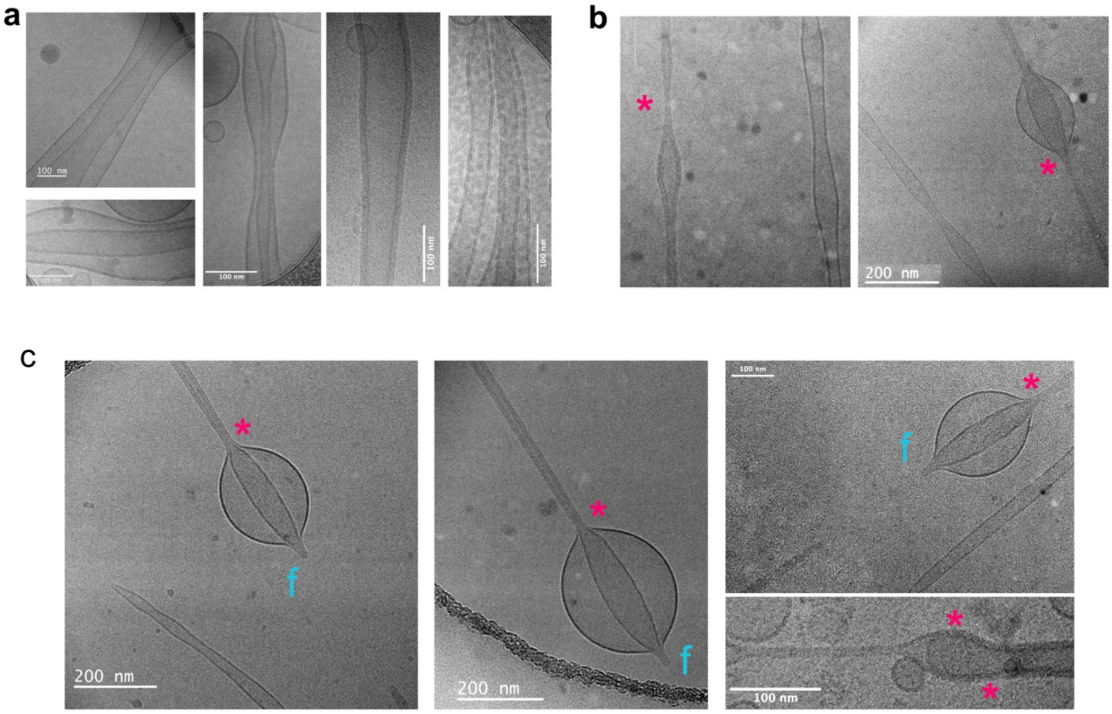

Supplementary Figure 2. CryoEM snapshots of DM tubes. Raw data for micrographs in Fig. 3 are shown. **a.** Collection of images of DM tubes with relatively low curvatures. **b.** DM tubes at high membrane constriction. Red asterisks indicate locations where the outer tube diameter is below that of the sum of two separate membranes. **c**. Fission of DM tubes. Red asterisks indicate locations where the outer tube diameter is below that of the sum of two separate membranes, while blue "f" indicates the fission of the DM tube.

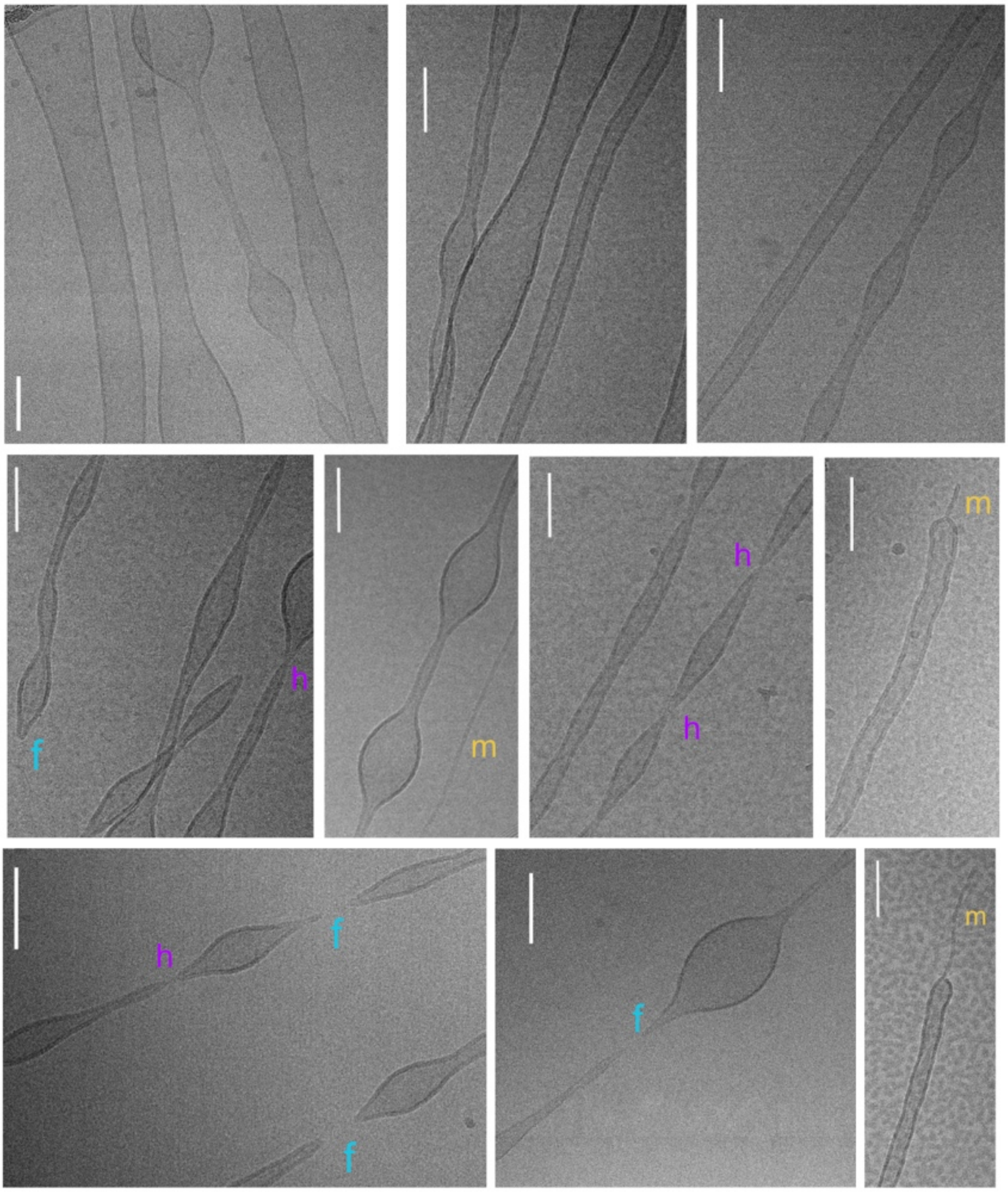

Supplementary Figure 3. CryoEM snapshots of SM tubes. A collection of micrographs shows examples of SM tubes in quasi-dynamic cryoEM settings. Blue “f” indicates fission events, magenta “h” indicates wormlike micelles (WLM) formation, while yellow “m” indicates an elongated WLM observed, capping the tubes upon fission. All scale bars are 100 nm.

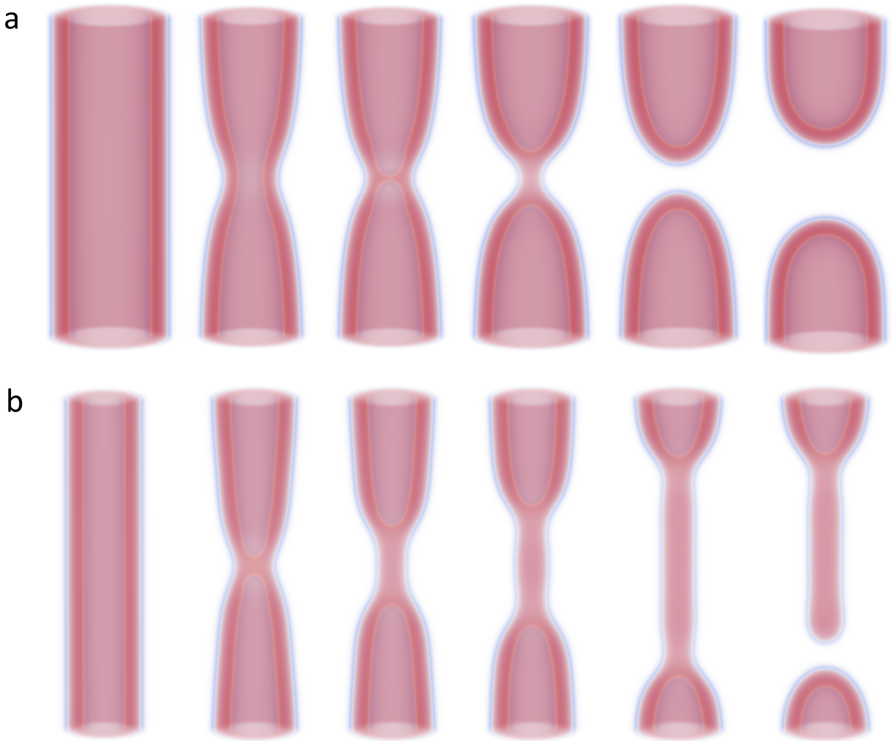

Supplementary Figure 4. Dependence of WLM extension on membrane tension. Lipid densities during the fission of SM tubes with membrane tensions of approx. 1 Dyn/cm (**a**) and 3 Dyn/cm (**b**). The tube first locally collapses, expelling the inner monolayer and creating a pair of capped tubes connected by a stalk (short WLM). The stalk may rupture right away, as in (a), or elongate into a long WLM before fission, as in (b). Which behavior is preferred depends on tension: At higher constriction (tension above ~2Dyn/cm) the WLM has a lower free energy than the SM tube, making collapse preferable.

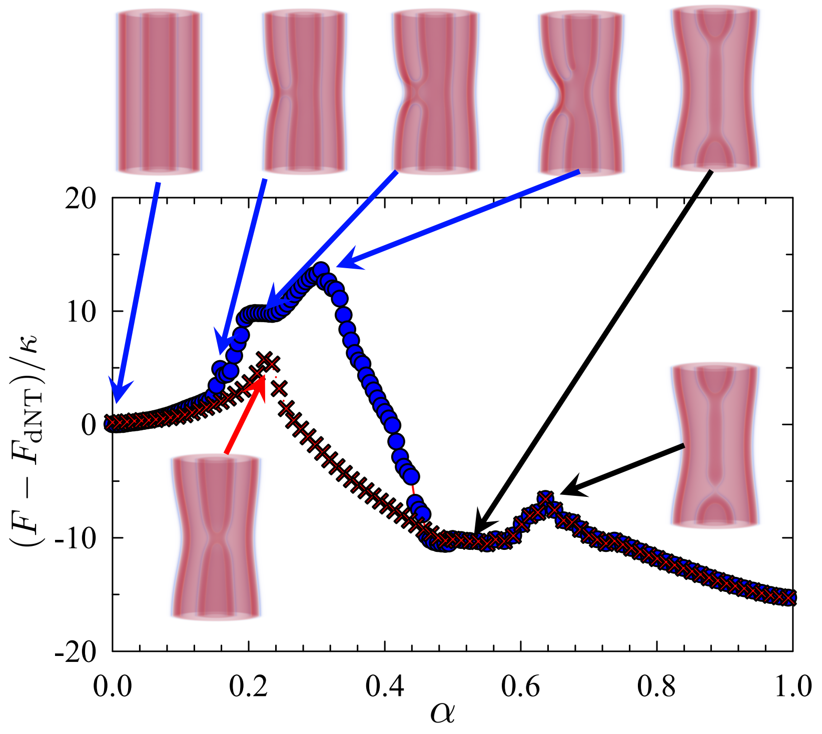

Supplementary Figure 5. DM fission pathways at high tension. The graph is analogous to Fig. 2b in the main text but at a tension of ~3 Dyn/cm, corresponding to an inner tube radius of ~ 4.5 nm. Crosses show the free energy along the canonical pathway, and circles show the hemifusion (HF) pathway. Images show key structures along the HF (blue circles) and canonical (red crosses) paths. This DM system will preferentially undergo fission via the classical pathway. For this highly constricted membrane tube, the barrier along the canonical pathway shrinks substantially, whereas the barrier to forming a stalk between the membranes remains approximately the same as for the tube with lower tension, described in the main text. The HF pathway is also further complicated by the extension of the WLM (see also Supplementary Figs. 4 and 6) and a new intermediate: a HF diaphragm, which becomes metastable for the constricted tubes (top middle). More detailed depictions are presented in Supplementary Fig. 6. The crossover in preference from the HF pathway to the canonical pathway is difficult to locate precisely due to the multi-step nature of the HF pathway and the changing nature of the multistep HF pathway (from one to two intermediates). While additional investigation is required to understand this complex behaviour, our data suggest that the crossover occurs when the inner-tube radius is around 6-7 nm. Source data are provided as a Source Data file.

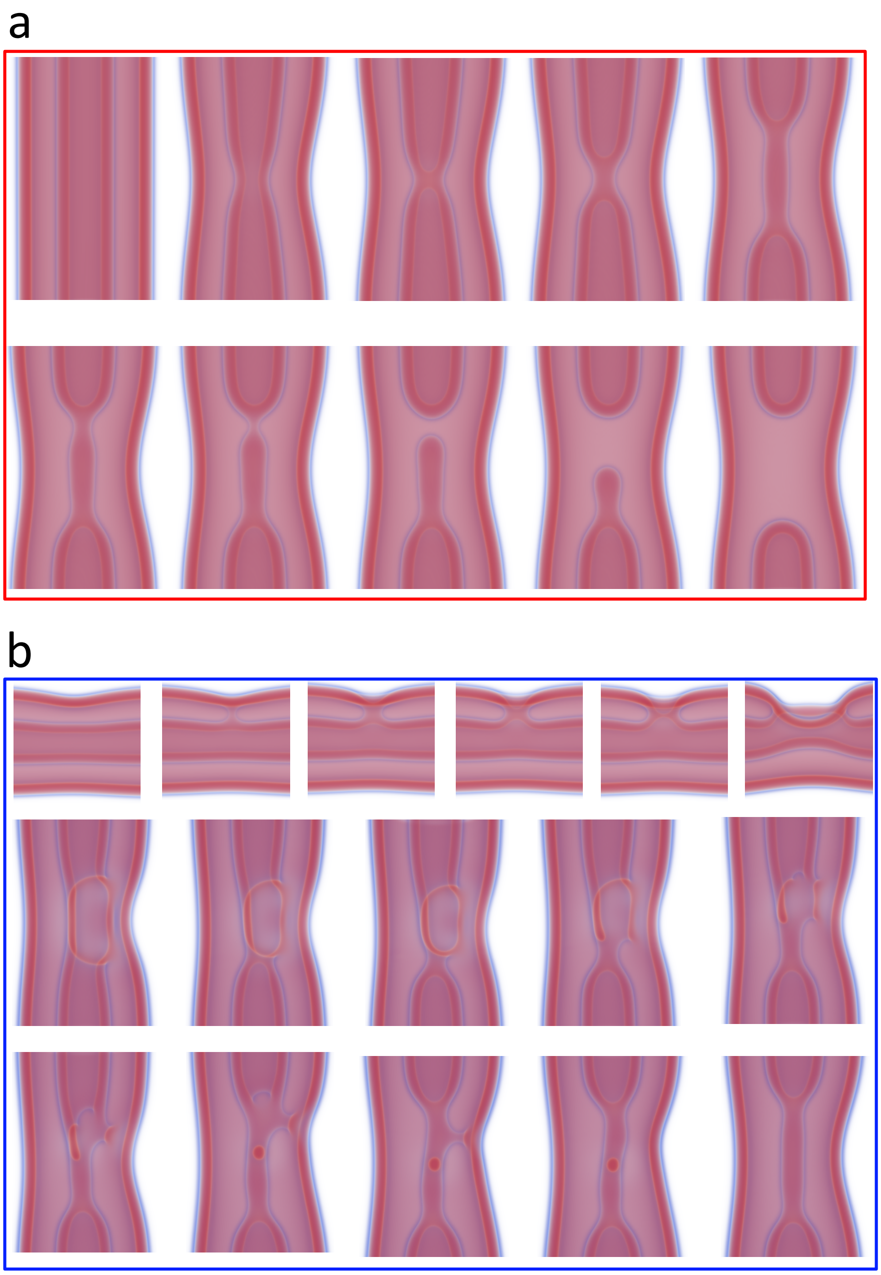

Supplementary Figure 6. Intermediate steps in the pathway presented in Supplementary Fig. 5. **a**. Direct inner-tube fission via a WLM intermediate. The inner tube locally constricts and forms a WLM that elongates; then, the WLM thins near a connection point, ruptures, and retracts, resulting in two capped tubes. **b**. Fission through the HF pathway. The inner and outer tubes first hemifuse. The HF catalyzes the collapse of the inner tube into a WLM, which ruptures as in a.

Supplementary Methods

Theory Overview

Biological bilayer membranes are formed by the self-assembly of lipid molecules in water. The lipids are amphiphilic, composed of a hydrophilic head group and a hydrophobic tail. We represent a lipid by an AB diblock copolymer composed of fN segments of type A (tail) and $(1-f)N$ segments of type B (head). The solvent, typically water, is represented as a short homopolymer composed of N_s segments of type B. The molecules are modeled as flexible Gaussian chains, where each segment has a statistical length, b, and volume, ρ^{-1}. The natural end-to-end length of a lipid (copolymer) is thus $R_0 = b\sqrt{N}$, which we use as our unit length.

The system of n_ℓ lipids and n_s solvent molecules is contained in a volume $V = (n_\ell N + n_s N_s)/\rho$. Throughout this work, we fix $N_s = N/10$ and $f = 0.8$. The repulsion between A and B segments is characterized by the Flory-Huggins interactions parameter, which we fix at $\chi N = 30$. Calculations are performed in the semi-grandcanonical ensemble, *i.e.* the numbers of each chemical species, n_ℓ and n_s, are not fixed. We rather constrain the total monomer number density, ρ, and the exchange chemical potential, $\mu = \mu_\ell - \mu_s$, which is the difference between the chemical potentials of lipids and solvent molecules.

Self-consistent Field Theory

The structure and thermodynamics of our lipid solution is calculated using self-consistent field theory (SCFT).[2,3] Molecules are modeled as Gaussian chains with the position along the molecule parameterized by s, where $0 < s < 1$ for the lipids and $0 < s < N_s/N$ for the solvent molecules. In order to calculate the statistics of the polymers, we need to calculate the partial partition function, or propagator, $q_T(\boldsymbol{r}, s)$, for the lipid ($T = \ell$) and solvent ($T = s$). This function gives the partition function for a portion of a chain from 0 to s, conditional on the segment at s being fixed at spatial position $\boldsymbol{r}$. The propagator satisfies

$$\frac{\partial}{\partial s} q_T(\boldsymbol{r}, s) = \left[\frac{R_0^2}{6} - w_\gamma(\boldsymbol{r})\right] q_T(\boldsymbol{r}, s) \quad (1)$$

where $w_\gamma(\boldsymbol{r})$ is the field felt by a γ-type segment: $\gamma = A$ for $s < f$ on the lipid and $\gamma = B$ for $s > f$ and for the homopolymer. $q_T(\mathbf{r}, s)$ is calculated by integrating Equation (1) with uniform initial conditions. The back propagator, $q_T^\dagger(\mathbf{r}, s)$ is similar but is solved with one side of Equation (1) multiplied by -1, as we are integrating from the other end of the molecule.

The propagator may be calculated using different modified diffusion equations to use alternate polymer models, for example worm-like or freely jointed chains. The Gaussian chain model has been the model of choice in previous SCFT membrane studies as it captures the chain connectivity and thus overall properties of the lipids, without requiring exorbitant computational costs. Owing to the universality of self-assembly in amphiphilic polymers and previous success using this model, we choose it to capture the behavior of membranes, and account for quantitative differences by matching the bending modulus of the SCFT model with that of real lipid systems.

The dimensionless concentrations of A and B segments are then given, respectively, by

$$\phi_A(\mathbf{r}) = z\int_0^f q_\ell(\mathbf{r},s)q_\ell^\dagger(\mathbf{r},s)ds\,,$$
$$\phi_B(\mathbf{r}) = z\int_f^1 q_\ell(\mathbf{r},s)q_\ell^\dagger(\mathbf{r},s)ds + \int_0^{N_s/N} q_s(\mathbf{r},s)q_s^\dagger(\mathbf{r},s)ds\,, \tag{2}$$

where the fugacity is $z \equiv \exp(\mu/k_BT)$. We also calculate the partition functions for the lipids and solvent molecules,

$$Q_\ell = \int q_\ell(\mathbf{r},1)d\mathbf{r},$$
$$Q_s = \int q_s\left(\mathbf{r},\frac{N_s}{N}\right)d\mathbf{r}. \tag{3}$$

It is convenient to write the fields in terms of a composition field, $w_-(\mathbf{r}) = \frac{w_A(\mathbf{r})-w_B(\mathbf{r})}{2}$ and a pressure field, $w_+(\mathbf{r}) = \frac{w_A(\mathbf{r})+w_B(\mathbf{r})}{2}$, which are conjugate to the composition, $\phi_-(\mathbf{r}) = \phi_A(\mathbf{r}) - \phi_B(\mathbf{r})$, and total concentration $\phi_+(\mathbf{r}) = \phi_A(\mathbf{r}) + \phi_B(\mathbf{r})$, respectively. In the usual manner of SCFT, we calculate the following quantities,

$$\Delta w_-(\mathbf{r}) = \chi N\phi_-(\mathbf{r}) - 2w_-(\mathbf{r}),$$
$$\Delta w_+(\mathbf{r}) = \chi N(\phi_+(\mathbf{r}) - 1), \tag{4}$$

which are essentially local chemical potentials acting on the composition and total concentration respectively. The SCFT algorithm involves initializing the system with some initial fields, $w_-(\boldsymbol{r})$and $w_+(\boldsymbol{r})$, and adjusting them until $\Delta w_-(\boldsymbol{r}) = \Delta w_+(\boldsymbol{r}) = 0$. Configurations satisfying this condition are stable or metastable configurations. In our numerical calculations, we update the fields until we reach a root mean-squared error of $\lesssim 10^{-4}$.

Once the configuration has been determined, the grandcanonical free energy, F, is then calculated using

$$\frac{F}{\sqrt{\bar{N}}k_BT} = -Q_s - zQ_\ell + \int \left(\frac{w_-^2}{\chi N} - w_+(\mathbf{r})\right)d\mathbf{r} \tag{5}$$

where $\bar{N} = (\rho R_0^3/N)^2$ is the invariant polymerization index. The Q terms give us the free energy of non-interacting polymers subject to the fields described above. Since the fields represent interactions with the other polymers, this double-counts the interaction energy between polymers. The integral over the fields subtracts the double-counted energy. Once we have obtained stable or metastable structures, we compare the free energies in order to determine the relative stability of different phases. SCFT is a mean-field theory and ignores compositional fluctuations, however, fluctuation corrections are known to only be important for weakly-segregated systems, which lipid membranes are not.

In our numerical implementation, we typically use 60 steps along the lipids and 6 along the homopolymers (solvent). Steps along the chain (integrating Equation 1) are done using a pseudospectral method. The field and concentrations are represented on a grid, with a grid spacing of $0.08R_0$, which we find to be sufficient to avoid discretization effects. We typically use a 3D grid of dimensions $16R_0 \times 12R_0 \times 12R_0$, however, for the cylindrically symmetric phases, we can greatly decrease computation time by using a 2D grid and a cylindrical coordinate system.

String Method

This work focuses on the transformation pathways between stable and metastable structures, and as such requires a method of determining how system configurations change when transforming from one configuration to another. We use the string method to find the Minimum Free-Energy Path (MFEP), connecting configurations.[4–9] The MFEP provides a thermodynamically reversible estimate for the most probable pathway between metastable states.

The string method involves considering m configurations indexed by i in the range $1 \leq i \leq m$. This is a discretization along the reaction coordinate, α, into m points. We label the fields $w_-^{(i)}$ and $w_+^{(i)}$, and concentrations $\phi_-^{(i)}$ and $\phi_+^{(i)}$. α is determined by calculating the Euclidean distance between adjacent points on the string and normalizing the total string length to 1, *i.e.*

$$\alpha_{i+1} - \alpha_i = \frac{\int [w_-^{(i+1)}(\boldsymbol{r}) - w_-^{(i)}(\boldsymbol{r})]^2 dr}{\sum_{j=1}^{m-1} \int [w_-^{(j+1)}(\boldsymbol{r}) - w_-^{(j)}(\boldsymbol{r})]^2 dr} \tag{6}$$

The ends of the string, $i = 1$ and $i = m$, are updated as described previously and relax into stable or metastable configurations. The intermediate points are updated similarly, but only the portion of Equation (4) perpendicular to the string is used (this is clarified below), and the configurations are shifted along the string, using a cubic interpolation, so that they remain equidistant in α, i.e. $\alpha_i = (i-1)/(m-1)$.

The perpendicular component of Equation (4) is calculated by subtracting the component that is parallel to the string,

$$\Delta w_-^{\perp}(\boldsymbol{r}, \alpha) = \Delta w_-(\boldsymbol{r}, \alpha) - \Delta w_-^{\parallel}(\boldsymbol{r}, \alpha) \tag{7}$$

where the field, $w_-(\boldsymbol{r})$, which is represented on a discrete grid of M points, is treated as an M-dimensional vector and "perpendicular" and "parallel" are defined in this M-dimensional space, as is the dot product below. The parallel component is

$$\Delta w_-^{\parallel}(\boldsymbol{r}, \alpha) = \hat{w}(\boldsymbol{r}, \alpha) \left(\Delta w_-(\boldsymbol{r}, \alpha) \cdot \hat{w}(\boldsymbol{r}, \alpha) \right) \tag{8}$$

where

$$\hat{w}(\boldsymbol{r}, \alpha) = \frac{\mathrm{d}w_-(\boldsymbol{r}, \alpha)}{\mathrm{d}\alpha} \left| \frac{\mathrm{d}w_-(\boldsymbol{r}, \alpha)}{\mathrm{d}\alpha} \right|^{-1} \tag{9}$$

is the unit vector pointing parallel to the string. The term in parentheses in Equation (8) is the magnitude of the component of $\Delta w_-(\boldsymbol{r}, \alpha)$ pointing parallel to the string.

In addition to making the update more direct, as it only acts perpendicular to the string, this modification allows the update to monitor the degree of convergence more accurately. The completely converged string satisfies $\Delta w_-^{\perp}(\boldsymbol{r}, \alpha) = 0$; however, due to the computationally intensive nature of these calculations, we are typically satisfied with a root-mean-squared error of $\lesssim 10^{-3}$.

References

1. Martinez Galvez, J. M., Garcia-Hernando, M., Benito-Lopez, F., Basabe-Desmonts, L. & Shnyrova, A. V. Microfluidic chip with pillar arrays for controlled production and observation of lipid membrane nanotubes. *Lab Chip* (2020) doi:10.1039/D0LC00451K.
2. Fredrickson, G. *The Equilibrium Theory of Inhomogeneous Polymers (International Series of Monographs on Physics)*. (Oxford University Press, USA, 2006).
3. Self-consistent field theory and its applications. in *Soft Matter, Volume 1: Polymer Melts and Mixtures* (eds. Gompper, G. & Schick, M.) vol. 1 87–178 (Soft Matter, Volume 1: Polymer Melts and Mixtures, 2006).
4. Maragliano, L., Fischer, A., Vanden-Eijnden, E. & Ciccotti, G. String method in collective variables: Minimum free energy paths and isocommittor surfaces. *J Chem Phys* **125**, 24106 (2006).
5. E, W., Ren, W. & Vanden-Eijnden, E. Simplified and improved string method for computing the minimum energy paths in barrier-crossing events. *J Chem Phys* **126**, 164103 (2007).
6. E, W., Ren, W. & Vanden-Eijnden, E. String method for the study of rare events. *Phys Rev B* **66**, 251 (2002).
7. E, W., Ren, W. & Vanden-Eijnden, E. Finite Temperature String Method for the Study of Rare Events. *J Phys Chem B* **109**, 6688–6693 (2005).
8. E., W. & Vanden-Eijnden, E. Towards a Theory of Transition Paths. *J Stat Phys* **123**, 503 (2006).
9. E, W. & Vanden-Eijnden, E. Transition-Path Theory and Path-Finding Algorithms for the Study of Rare Events. *Annu Rev Phys Chem* **61**, 391–420 (2010).
