# Supplementary material for: Membrane fission via transmembrane contact
Source: Nat Commun. 2024 Mar 30;15:2793. doi: 10.1038/s41467-024-47122-w (PMC10981662; doi:10.1038/s41467-024-47122-w)
Supplement: Supplementary file 3 — Description of Additional Supplementary Files [file 41467_2024_47122_MOESM3_ESM.pdf]

Description of Additional Supplementary Files

File Name: Supplementary Movie 1
Description: DM tube constriction and fission under osmotic stress, corresponding to Fig. 1b. RhPE fluorescence is shown.

File Name: Supplementary Movie 2
Description: Video of the HF pathway from SCFT and the string method, as depicted in Fig. 2b-c.

File Name: Supplementary Movie 3
Description: Real time imaging of constriction and fission of membrane tubes induced by hydrodynamic flow applied in a microfluidic chamber by infusion of buffer at 15 L/min rate. The experiment mimics the blotting conditions in the quasi-dynamic cryoEM experiments. RhPE fluorescence is shown. Progressive loss in intensity indicates membrane tubes thinning. Red arrow indicates the moment of scission of one of the tubes.
